# A simplified, fluoroless workflow for atrial fibrillation pulsed field electroporation

**DOI:** 10.1002/joa3.70057

**Published:** 2025-04-02

**Authors:** William K. Chan, Umjeet S. Jolly

**Affiliations:** ^1^ Heart Rhythm Program St. Mary's General Hospital Kitchener Ontario Canada; ^2^ Department of Medicine McMaster University Hamilton Ontario Canada

## Abstract

We detail a streamlined workflow using intracardiac echocardiography that does not require internal jugular venous access, pre‐procedural imaging, or additional multielectrode catheters. Overall, this may improve the efficiency and reduce the overall costs of PFA with a variable loop circular mapping catheter without necessarily compromising on efficacy or safety.

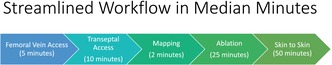

We read with interest about the article by Hirata and colleagues on a zero fluoroscopy workflow using a variable loop circular mapping catheter (VLCC) integrated in a 3D electro‐anatomical mapping system.^1^ The authors should be congratulated on their diligence and commitment to a thorough workflow. Interestingly, the authors used several catheters in their approach through at least two femoral vein accesses and one internal jugular vein access. While it is convenient to have additional catheters, we argue that they are not necessary for the workflow, and the incremental cost of consumables may affect the uptake of this technology into regular practice, especially in resource‐constrained environments.^2^ In addition, multiple catheter exchanges can increase the risk of cerebrovascular events by way of introducing micro‐bubbles. We share our initial, single‐centered experience involving 11 patients with a streamlined workflow using single‐sided femoral venous access.

All procedures were performed under general anesthesia to prevent map shifts from patient movements. Left atrial thrombus risk was mitigated with pre‐procedural imaging such as computed tomography or transesophageal echocardiography, and the procedure was performed on continuous anticoagulation held <24 h. No invasive arterial hemodynamic monitoring was used. Ultrasound‐guided access was obtained in the right femoral vein only with two sheaths—one for the intracardiac echocardiography (ICE) catheter (SOUNDSTAR™, Biosense Webster) and the other for the steerable VIZIGO™ sheath (Biosense Webster). Once both sheaths were in the vein, a heparin IV bolus was delivered to target ACT >350 s, followed shortly by glycopyrrolate 0.2–0.4 mg IV to blunt any anticipated vagal responses from ablation. 3D geometry of the pulmonary veins, left atrium, and coumadin ridge was reconstructed with the CARTOSOUND™ FAM Module powered by artificial intelligence, which eliminates the need for manual contouring.^3^ An anterior‐inferior, single transseptal puncture was performed with a transseptal needle introduced through the VIZIGO™ sheath under direct ICE guidance. Once the VIZIGO™ sheath was advanced into the left atrium through the fossa ovalis, the multi‐electrode, irrigated, pulsed field electroporation VLCC was introduced through this sheath without need for further catheter exchanges. A base impedance map was built through a process in which impedance and location measurements were accumulated as the VLCC was maneuvered in the left atrium for the tissue proximity indication (TPI).^4^ Additional fast anatomic mapping of the left atrium was not necessarily required if the geometry created by the CARTOSOUND™ FAM Module was felt to be complete by the operator.

Pulmonary vein isolation (PVI) was performed according to the standard workflow recommendations of four ablations per pulmonary vein (PV)—two to the ostial PV with rotation after the first ablation (to ensure coverage of the gap between electrodes 1 and 10), and two to the antral PV with again rotation after the first ablation using the TRUPULSE™ Generator (Biosense Webster).^5^ Each ablation consisted of three applications of energy with a 10‐s delay between applications using short‐duration, high voltage, bipolar biphasic pulses of 1800 V. Therefore, a minimum of 16 ablations were delivered for all the PV's, which represents 48 applications in total. Additional circumferential or segmental ablation was performed at the roof, left ridge, and/or right carina depending on tissue contact as indicated by TPI‐positivity in all ablation electrodes. Tissue‐catheter contact was also assessed under direct visualization with ICE in the right atrium. All PV's were targeted sequentially starting from the left upper, left lower, right lower, and right upper pulmonary veins. All PV's were acutely isolated showing entrance block without a waiting period, and final re‐mapping was performed at the discretion of the operators. No additional ablation beyond the PV's was performed. At the end of the procedure, pericardial effusion was ruled out with ICE and a single figure of 8 suture/stopcock was used for hemostasis. All patients were discharged on the same day.

Median times were reported for the following:

- Femoral vein puncture to left atrial access (15 min)
- Mapping time (2 min)
- Ablation time (7 min) and ablation duration (25 min)
- Left atrial dwell time (29 min)
- Total skin‐to‐skin time (50 min)

A median of 19 ablations was delivered with a total of 57 applications. Complete PVI was achieved in 100% of patients with no additional radio‐frequency ablation required. There were no major acute complications and no postprocedural atrial fibrillation recurrences to date.

Our streamlined workflow did not require internal jugular vein access, pre‐procedural imaging, left atrial matrix, or additional multi‐electrode catheters that would necessitate frequent catheter‐cable switching. Right atrial fast matrix was also not found to be helpful or needed. This fluoroless workflow requiring ICE has many benefits including single‐sided femoral access to improve early ambulation, zero catheter exchanges to minimize thrombo‐embolic risk, and minimal if any additional matrix required of the left atrium. Overall, this can improve efficiency and reduce the overall costs of the procedure without compromising on efficacy or safety. Future prospective studies are needed to demonstrate that this workflow can be easily and universally adopted for more widespread use of the pulsed field electroporation VLCC for PVI.

## CONFLICT OF INTEREST STATEMENT

Both authors received honorariums from Biosense Webster.

## ETHICS STATEMENT

Approval of the research protocol: Yes, as we need to uphold rigorous ethical standards for the research we consider for publication.
